# Effect of Bayesian-penalized likelihood reconstruction on [13N]-NH3 rest perfusion quantification

**DOI:** 10.1007/s12350-016-0554-8

**Published:** 2016-07-19

**Authors:** Jim O’ Doherty, Daniel R. McGowan, Carla Abreu, Sally Barrington

**Affiliations:** 1 PET Imaging Centre, Division of Imaging Sciences and Biomedical Engineering, King’s College London, King’s Health Partners, St. Thomas’ Hospital, 1st Floor, Lambeth Wing, London, SE1 7EH United Kingdom; 2 Department of Oncology, University of Oxford, Old Road Campus Research Building, Oxford, OX3 7DQ United Kingdom; 3 Radiation Physics and Protection, Churchill Hospital, Oxford University Hospitals NHS Foundation Trust, Oxford, OX3 7LE United Kingdom

**Keywords:** N-13 ammonia, PET, physics of imaging

## Abstract

**Objectives:**

Myocardial blood flow (MBF) imaging is used in patients with suspected cardiac sarcoidosis, and also in stress/rest studies. The accuracy of MBF is dependent on imaging parameters such as new reconstruction methodologies. In this work, we aim to assess the impact of a novel PET reconstruction algorithm (Bayesian-penalized likelihood—BPL) on the values determined from the calculation of [13N]-NH3 MBF values.

**Methods:**

Data from 21 patients undergoing rest MBF evaluation [13N]-NH3 as part of sarcoidosis imaging were retrospectively analyzed. Each scan was reconstructed with a range of BPL coefficients (1-500), and standard clinical FBP and OSEM reconstructions. MBF values were calculated via an automated software routine for all datasets.

**Results:**

Reconstruction of [13N]-NH3 dynamic data using the BPL, OSEM, or FBP reconstruction showed no quantitative differences for the calculation of territorial or global MBF (*P* = .97). Image noise was lower using OSEM or BPL reconstructions than FBP and noise from BPL reached levels seen in OSEM images between *B* = 300 and *B* = 400. Intrasubject differences between all reconstructions over all patients in respect of all cardiac territories showed a maximum coefficient of variation of 9.74%.

**Conclusion:**

Quantitation of MBF via kinetic modeling of cardiac rest MBF by [13N]-NH3 is minimally affected by the use of a BPL reconstruction technique, with BPL images presenting with less noise.

## Introduction

Quantitative myocardial blood flow (MBF) imaging with PET is utilized in many centers around the world in a clinical setting for investigation of the human coronary circulation.^1^-^3^ The technique allows for the quantitative assessment of the distribution of flow for delineation of the extent and severity of coronary artery diseases, microvascular function, as well as other conditions such as cardiac sarcoidosis. Radiological imaging detects cardiac involvement in almost 40% of patients with sarcoidosis and is more sensitive than ECG, Holter monitoring, and echocardiography,^4^ with a high sensitivity and specificity reported for [18F]-FDG PET of 89% and 78%, respectively.^5^ Quantification of MBF has been routinely used as an aid in suspected cardiac sarcoidosis in order to rule out coronary artery disease or to identify resting perfusion defects suggestive of inflammation-induced tissue damage.^6^ Studies have also shown that there is a characteristic uptake pattern in the myocardium of patients with active cardiac sarcoidosis.^7^ “Eyes-to-thighs” FDG PET-CT scanning is further used to detect any noncardiac granulomatous involvement (such as pulmonary), with atlases recently published showing the imaging features and patterns,8 and comprehensive imaging protocols have also recently been published.^6^

New commercial PET reconstruction algorithms are continuously being developed and investigatied, with their operation potentially affecting the final voxel values presented in PET images. Many new commercial methods include data corrections such as point-spread function modeling of the entire PET field of view aimed at improving spatial resolution during image reconstruction.^9^,^10^ One such example of this, and of interest to this work, is a new Bayesian-penalized likelihood (BPL) reconstruction algorithm developed by GE Healthcare (commercially named Q.Clear^®^). The technique involves point-spread-function modeling with noise modeling controlled through the use of a penalty term that penalizes image intensity differences between neighboring pixels. The penalty function is controlled by a unitless ‘beta value’ (henceforth called “*B*” in this work) which adjusts the strength of the regularizing term in the objective function of the reconstruction,^11^ and is the only input to the reconstruction algorithm rather than traditional iterations, subsets, and post-reconstruction smoothing filters of iterative OSEM algorithms. The algorithm is allowed to run to effective convergence, allowing for an improved quantitative accuracy of imaging rather than suspending the algorithm after a certain number of iterations to control the image noise.

Previous work has shown how BPL reconstruction algorithms provide improved signal-to-noise (SNRs) and signal-to-background ratios (SBRs) in the imaging of colorectal liver metastases,^12^ lung nodules,^13^ and mediastinal lymph nodes.^14^ Results of these studies showed significant increases in the average maximum standardized uptake value (SUV_max_) after BPL processing. Earlier work has focused on oncology [18F]-FDG PET and found a *B* value of 400 to be optimum.^15^ Image reconstruction has been shown to have an effect on resulting activity concentration in cardiac studies,^16^ and previous work has shown differences in [13N]-NH3 MBF of up to 11% between FBP and OSEM reconstructions.^17^ MBF has also been investigated in relation to technological changes such as 2D and 3D PET,^18^ and on different software packages for [13N]-NH3 MBF calculations,^19^ although to the best of our knowledge, no studies have yet investigated the use of new PSF reconstruction methods in dynamic [13N]-NH3 cardiac studies, or investigated the effects of different *B* values for these studies. The aim of this study was therefore to evaluate the quantitative effect on calculated MBF values of employing the BPL algorithm with different penalization factors on [13N]-NH3 images with a range of *B* values, compared to our imaging standard of FBP reconstruction.

## Methods

### Patients and Scanning

Clinical scans from 21 patients (8 female, 13 male, mean age 50.4 ± 12.5 years) imaged for suspected cardiac sarcoidosis comprising dynamic [13N]-NH3 scans were retrospectively analyzed. No selection criteria were applied to the patients. Only members of the clinical team, in compliance with the UK Data Protection Act, reviewed patient data, and consequently, specific Research Ethics Approval was not required. Patients were asked to fast for 12 hours as MBF imaging was combined with an [18F]-FDG viability imaging study, which required minimization of myocardial glucose uptake. All data were acquired on a GE Discovery 710 PET-CT scanner (GE Healthcare, Waukesha, USA) at Site 1 (St Thomas’ Hospital, London, UK). BPL reconstruction via Q.Clear was not available at Site 1, and thus, RAW PET sinograms and PET calibration files were sent to Site 2 (Oxford University Hospitals, Oxford, UK) for reconstruction as outlined below.

### [13N]-NH3 Imaging

A cine-CT was acquired (100 kVp, 10 mA, 0.5-second rotation, 5.5-seconds cine duration, and 40-mm collimation) for attenuation correction, and was reconstructed to 2.5-mm contiguous slices (704 images). ECG gating was not used for PET or CT imaging. Following the cine CT, patients were injected with an average activity concentration of 527 ± 24 MBq of [13N]-NH3, and 3D PET was acquired in listmode.

Attenuation correction of [13N]-NH3 PET images was performed using an average of the acquired cine CT, a well-published method to correct for potential respiratory registration artifacts in [13N]-NH3 cardiac imaging by matching CT and PET temporal resolutions.^20^ Due to enzymatic conversion of ammonia to glutamate, the final 20 minutes’ duration of the 26 minutes acquisition was used to reconstruct a single static frame of 47 slices (not part of this analysis), which was then used to determine the registration vector between the PET and the average cine CT using ACQC (Attenuation Correction Quality Control) software present on the PET-CT scanner. CT and PET images were manually aligned to provide the best visual registration and the resulting shift vector was then applied to the reconstruction of the dynamic data. An experienced clinician reviewed the images of each registration as part of a standard clinical protocol.

List-mode PET data were re-binned to 12 × 10 seconds, 6 × 20 seconds, 2 × 1 minutes and 1 × 20 minutes. Only the first 6 minutes’ duration of data was used for MBF calculation. For clinical reporting of MBF, images were reconstructed at Site 1 using a filtered back-projection (FBP) algorithm with a 6.4-mm post-smoothing filter (enhanced Hanning). We also reconstructed a time-of-flight OSEM dataset (2 iterations, 24 subsets, 6.4-mm Gaussian filter) for comparison. RAW PET sinograms and average cine CT datasets were transferred to Site 2, who then reconstructed the sinograms with the same FBP and OSEM reconstruction parameters for quality assurance (to ensure that MBF values were equivalent from both sites), as well as performing BPL reconstructions with values of *B* = 1, 50, 100, 200, 300, 400, and 500 (guided by earlier work on [18F]-FDG imaging). Site 2 employed the normalization, and dose calibrator files from Site 1 that were required for image reconstruction. Reconstructed images were then sent to Site 1 for final MBF analysis.

### Image and Data Analysis

Myocardial blood flow (MBF) values were calculated from the reconstructed dynamic [13N]-NH3 data using SiemensMBF software (Siemens Medical Solutions, Erlangen, Germany) on a Syngo workstation. The software employs a 2-tissue compartment [13N]-NH3 kinetic model with four parameters (1 vascular volume and 3 transport coefficients) describing extraction and retention of [13N]-ammonia in myocardial tissue.^21^ After loading data into the software, the program automatically performed segmentations of myocardial walls and also placed a VOI in the left ventricle for determination of an image-derived input function (IDIF). No manual adjustments were made to the automatically segmented volumes. Values of MBF (mL·minute^−1^·g^−1^) were assigned to the standardized American Heart Association (AHA) 17-segment model, and vascular territories were defined based on the standard division of the polar map.^22^

## Results

**Figure 1:**
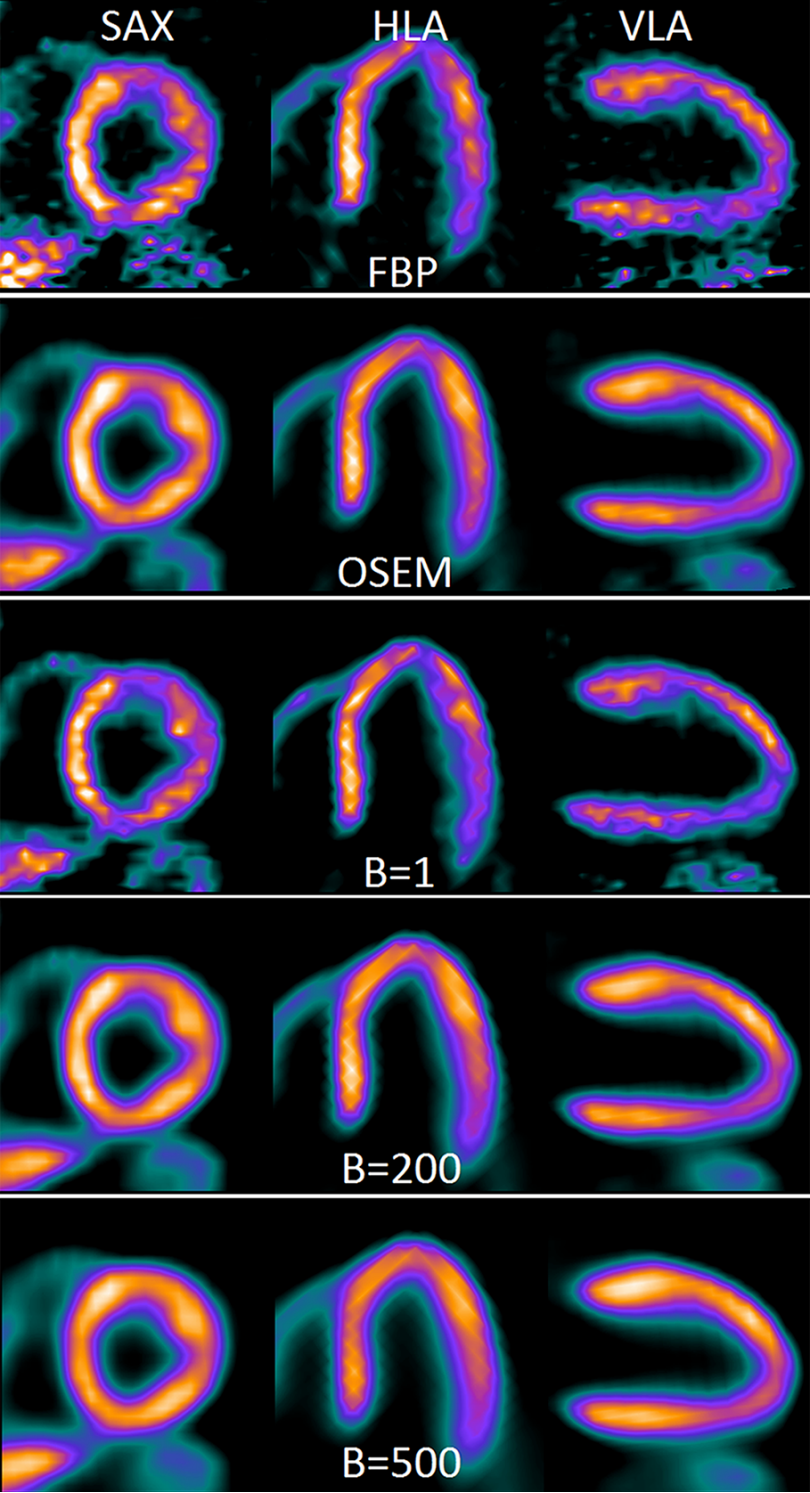
Slices from a final 20 minutes’ duration image detailing comparison of the reconstructions performed including FBP, OSEM (2i,24s) and BPL with beta values of 1, 200 and 500. Images are displayed on the same window widths and levels. A higher level of smoothing with higher *B* values can be observed

**Figure 2:**
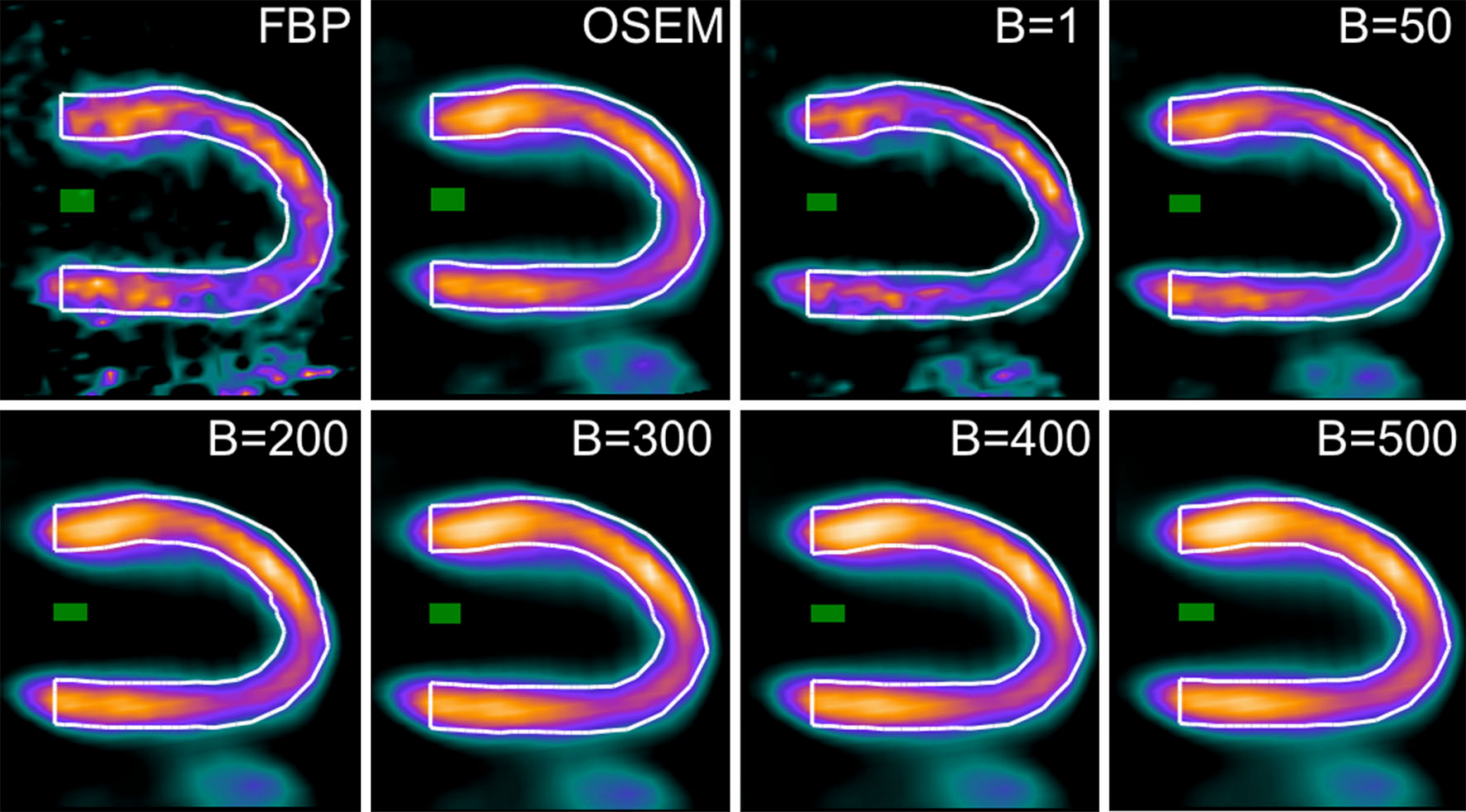
Automatic image segmentation of [13N]-NH3 images showing successful segmentation for all reconstructions. An automatically drawn 1 × 1 × 1 cm volume at the mitral valve plane represents the position of the left ventricular image-derived input function (IDIF). Images are displayed on the same window width and level

Figure 1 shows the same single short axis, vertical long axis and horizontal long axis slice from the summed 20-minute images from FBP, and also from the increasing *B* value from BPL reconstructions. Increased levels of smoothing can be seen with the increasing *B* value. Figure 2 demonstrates example results of the automated segmentation procedure on each reconstruction, showing successful segmentation over the entire range of *B* values independent of the levels of smoothing resulting from the reconstruction.

**Figure 3:**
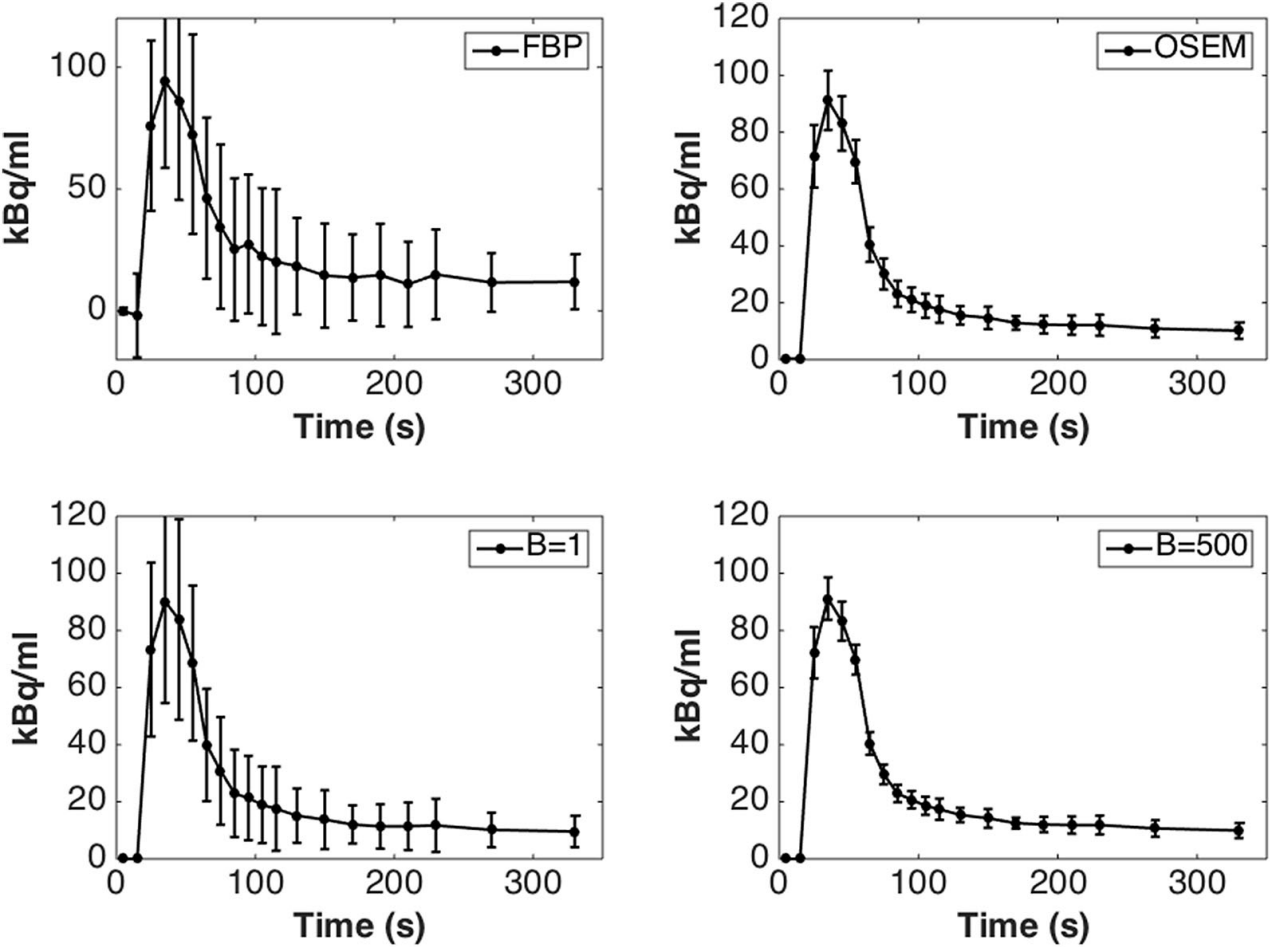
Comparison of blood input functions automatically drawn from the left ventricle of a single subject for four individual reconstructions (FBP, OSEM, BPL *B* = 1 and *B* = 500). A decrease in the noise (assessed by standard deviation of the mean of VOI) can be observed with the increasing *B* value

**Figure 4:**
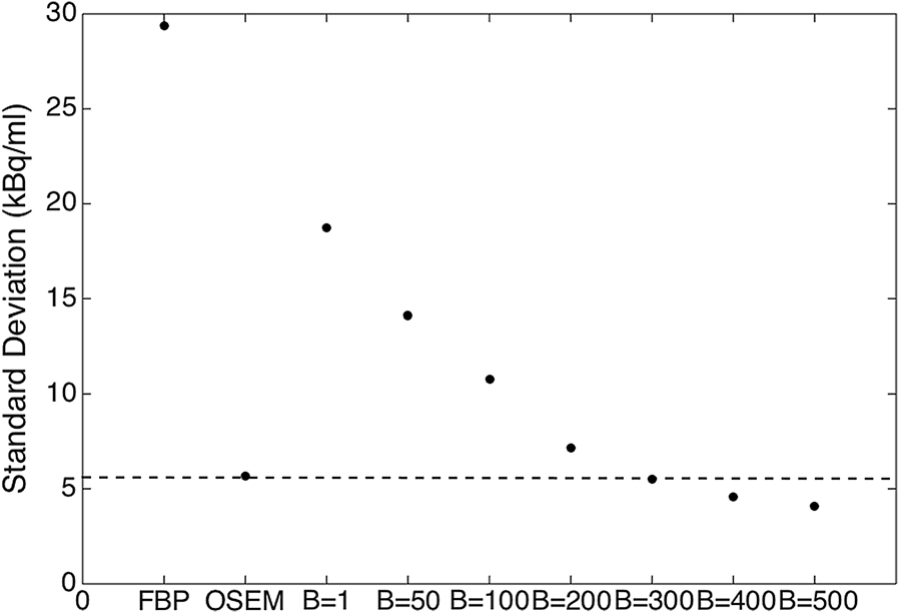
Example of a reduction in the standard deviation averaged across all 10-second time frames of an IDIF with the increasing *B* value compared to standard FBP and OSEM reconstructions. The image noise can be observed to be at OSEM levels at approximately *B* = 300

**Figure 5:**
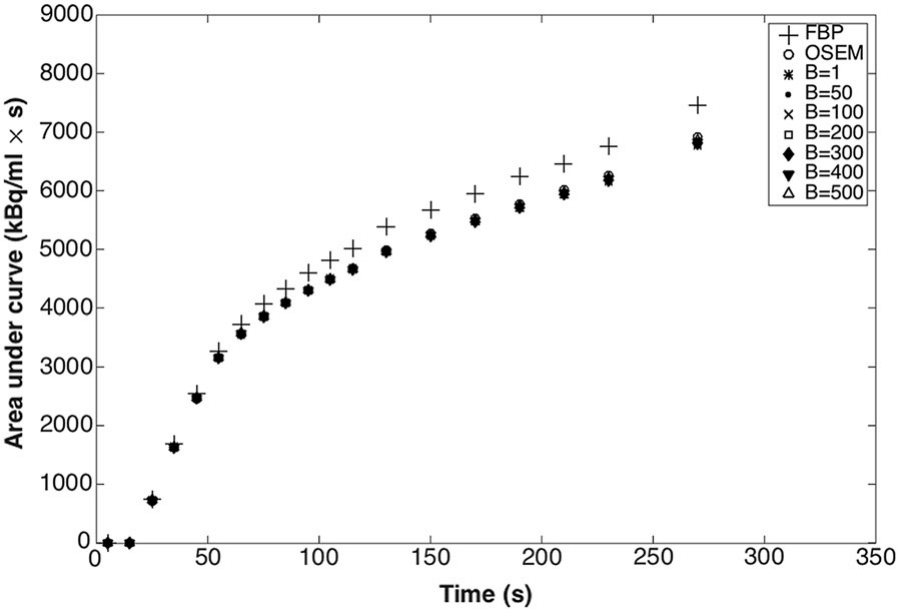
Example of cumulative area under the IDIF for each reconstruction for a single patient using the mean activity concentrations. The maximum difference in total area from the standard of FBP is 12.2% (*B* = 50—and the largest in the entire study), and the minimum is 6.1% (OSEM). The maximum difference in AUC for each patient did not correspond with any specific reconstructed algorithm

Examples of the image IDIFs required to drive the kinetic model used in quantification software are shown in Figure 3, where the effects of the reconstruction algorithms on image noise (assessed by ±1 standard deviation of the mean of the region of interest) can clearly be observed. Noise was seen to decrease with the increasing *B* value, as shown by example in Figure 4, where the standard deviation of a single IDIF has been averaged (using the 12 × 10 seconds frames only) for a single patient. When repeated for all patients, OSEM levels of noise in IDIF and myocardial uptake curves were observed between *B* = 300 and *B* = 400. We used FBP reconstruction as a gold standard, as is common in dynamic cardiac PET imaging utilizing kinetic modeling, due to low system bias, reliable quantitative capabilities and ability to cater for rapid time varying changes in activity concentration.^16^ Using this methodology, calculation of the area under the curve (AUC—a critical parameter for performing PET compartmental kinetic modeling^23^) of the input function for all reconstructions (FBP, OSEM, *B* = 1, 50, 100, 200, 300, 400, 500) showed a maximum difference over all patients of 12.2% lower with *B* = 50. The cumulative AUC is shown in Figure 5. A minimum difference over all patients of 6.1% lower than FBP with *B* = 400 in the case shown was determined. Although FBP showed a consistently higher AUC, maximum and minimum differences in AUC between FBP and BPL algorithms did not correspond with any specific *B* value.

On performing FBP and OSEM reconstructions at Site 1 and independently for the same reconstructions at Site 2, automated analysis resulted in identical MBF values. This was an important step showing that RAW PET data was transferrable, and reconstruction at Site 2 was equivalent in terms of scanner normalization files and corrections, and therefore, the only changes affecting the reconstruction was the implementation of the BPL algorithm.

**Figure 6:**
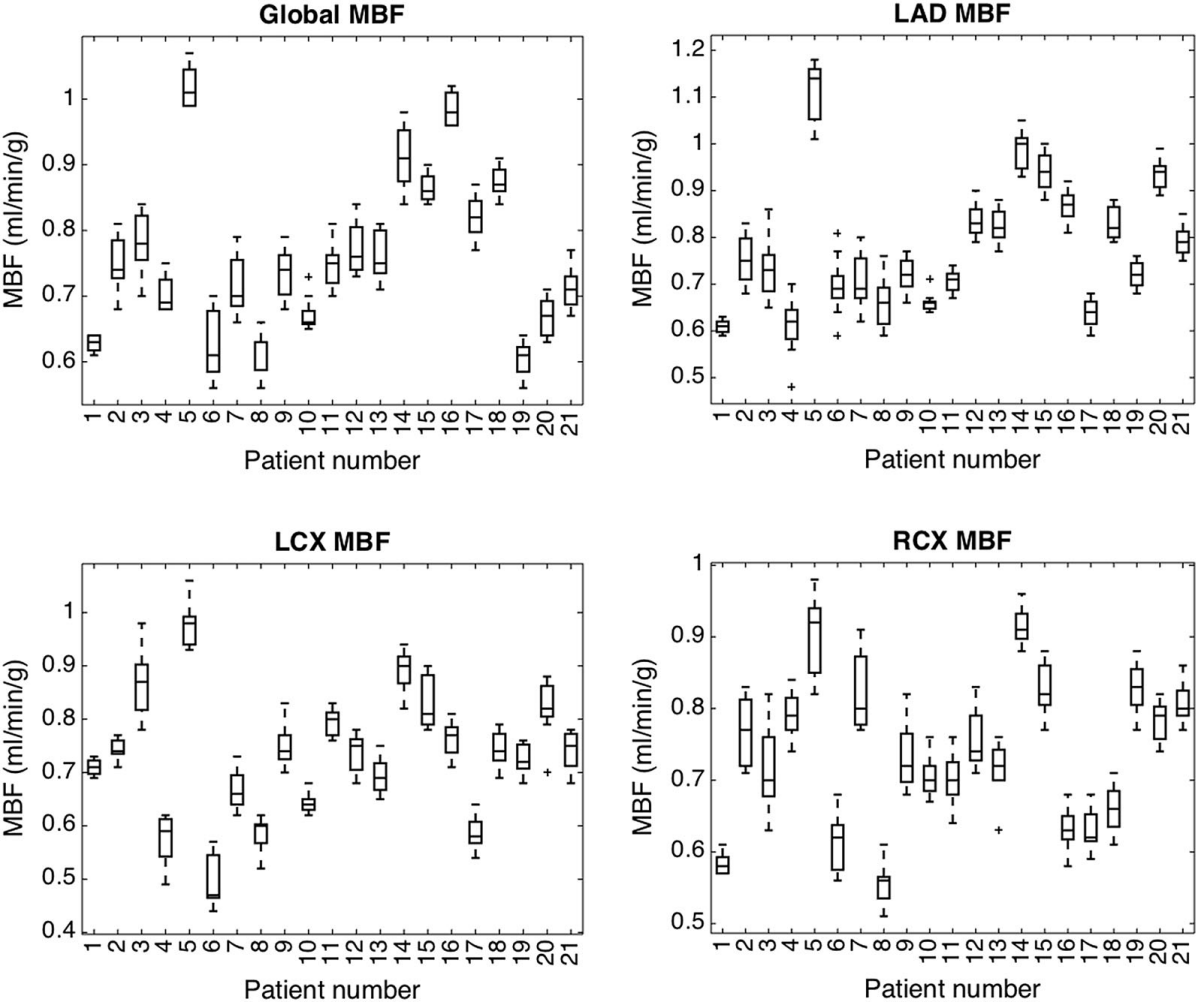
Global and territorial MBF results from all patients (1-10) with all reconstructions. The resulting MBF values for each patient are closely correlated with a maximum coefficient of variation of 9.74% over all patients and territories

MBF values for each vascular cardiac territory, left anterior descending (LAD), left circumflex (LCX), and right coronary artery (RCX) as well as a global MBF values were automatically computed by the kinetic modeling software. Overall, MBF from each patient using all reconstructions were closely correlated, with a range of coefficient of variation (CoV) from 1.95% (min) − 9.74% (max) over all vascular territories and also globally. A box-plot of all MBF values is shown in Figure 6. One-way ANOVA results comparing a single parameter (image reconstruction) in each vascular territory (LAD, LCX, and RCX) showed *P* > .95 for MBF values in all vascular territories, as well as in the global MBF, indicating that MBF values from all reconstructions for each patient were statistically equivalent. There were also no clearly defined bias trends resulting from any of the reconstructions.

We reconstructed and analyzed all images at a 128 matrix as part of the standard clinical protocol, which enabled a high speed of reconstructing 21 dynamic and many static datasets for each patient. However, we reconstructed all data for five of the dynamic [13N]-NH3 studies (selected at random) using 256 matrix size in order to investigate any effect on MBF, which produced a maximum difference in global MBF of 3.9% over all 5 patients (2.4% in LAD, 2.8% in LCX, and 3.1% in RCX over all five datasets, respectively). Thus, the matrix size of 128 has only a minor effect of the resulting MBF values. None of the five patient datasets showed any large change in MBF values on the higher matrix size, either for vascular territory or globally.

## Discussion

We have provided to the best of the authors’ knowledge, the first comparisons of a new fully converged BPL reconstruction algorithm on [13N]-NH3 cardiac MBF values, showing that MBF quantification of the PET data is not significantly affected over the range of penalization factors of *B* = 1 to *B* = 500 in BPL reconstruction. Images such as those shown in Figure 1 show increased smoothing at higher *B* values. As noted in previous work, due to the higher regularization penalty factor, images will appear smoother at the expense of causing blurring of the reconstructed images.^15^ However, as shown in Figure 2, the automatic segmentation routine was still able to reproducibly segment the myocardium from the images throughout the range of examined *B* values. The effects of the increased image noise of the IDIF as assessed by ±1 standard deviation of the values within the VOI shown in Figure 3 shows that although the mean IDIF is consistent over the range of *B* values, the standard deviation on each point decreases with the increasing *B* value. Similar noise relationships with the increasing *B* values were obtained for the myocardial uptake curves (not shown).

In our experience of using a commercially produced MBF quantitation program, the software reports an error value, although it is the error related to the fitting of MBF parameters resulting from the kinetic modeling process (K_1_), and is not inclusive of the noise in the IDIF or myocardial uptake curve. As shown in Figures 3 and 4, the effects of reconstruction can cause a large amount of noise in the resulting VOIs, and therefore should be accounted for by any analysis software. However, as shown by Figures 5 and 6, accounting for only the average activity concentration and no associated noise in the VOI, both the area under the IDIF and the resulting global and territorial MBF values do not experience significant changes with the increasing *B* value. For the patient with a maximum AUC difference of 12.2% compared to standard FBP reconstruction, this difference led to a difference of 6.3% in estimation of MBF in the LCX territory (0.63 and 0.59 mL·minute^−1^·g^−1^ for FBP and *B* = 400, respectively).

Our cohort of potential sarcoidosis patients did not represent an optimal cohort to assess the BPL reconstructions in [18F]-FDG viability imaging due to the intentional suppression of physiological [18F]-FDG uptake in the heart in order to minimize false-positive results. In this state, free fatty acids account for up to 90% of oxygen consumption of normal myocytes,^7^ and this technique in patients without cardiac sarcoid involvement produces images primarily of the blood pool, with poor myocardial uptake of FDG, except in cases where sarcoidosis was present, or in patients with suboptimal preparation. However, as cardiac [18F]-FDG imaging is only performed normally for sarcoidosis purposes at our center, patients undergoing viability imaging with [18F]-FDG for other purposes (such as coronary artery disease) could be used to investigate if and how the BPL reconstruction affects the uptake pattern of the [18F]-FDG.

As is customary in clinical cardiac studies, caution should be taken that PET data is attenuation corrected and respiratory corrected as appropriate, as inaccurate attenuation correction has been known to affect quantitation of cardiac PET studies.^20^,^24^ This study focused on clinically acquired data attenuation corrected with an average of a free-breathing cine CT acquisition as recommended in ASNC guidelines,^25^ however, there are many other strategies for providing AC and also respiratory gating^26^ with comparisons of methods being carried out.^20^,^27^ In our work, all data originated from the same raw sinograms, and thus, any motion affecting imaging would affect all reconstructions in the same manner, and hence it is the relative difference in MBF that was of interest. However, the use of a standardized dynamic test methodology to examine differences in reconstruction while remove the confounding effects of motion artifacts, and allowing a focus purely on the kinetic analysis would be a welcome addition to accurately clarify a comparison of reconstructions. Investigations with the use of a simplistic nonmoving cardiac perfusion phantom have recently been performed in MR imaging, and would provide a useful standard by which to compare reconstructions and benchmark kinetic modeling software analysis routines free from the considerations of motion artifacts.^28^ A further potential source of error is the scanner being affected by the high count rate due to all of the [13N]-NH3 activity being in the field of view at the same time. This would potentially increase the dead time of some of the PET detectors during the initial frames of imaging, affecting the input function used to drive the kinetic model.^29^ Although this was not explicitly accounted for in this work, dead time would have affected all of the reconstructions in the same manner, and therefore was disregarded for this analysis.

Our study represents preliminary data investigating this novel reconstruction algorithm, and should be further explored with a larger dataset. However, multi-institutional comparison of PET MBF studies remain limited by differences in tracers, kinetic models, technical methodology, image analysis software, and pharmacological vasodilating agents.^1^,^3^ For example, the reported PET stress MBFs in normal individuals vary from 1.86 ± 0.27 to 5.05 ± 0.90 mL·g^−1^·minute^−1^, with a 27% weighted average coefficient of variation for single measurements.^30^ Some standardization of MBF quantification has recently been performed by way of comparing image analysis software with [82Rb]-Cl^31^ or [13N]-NH3 data^19^ acquired at a single site. These studies show good correlation between the MBF values resulting from different analysis packages. Options for improving methodological standardization such as image reconstruction deserve careful study and may prove an important factor of the overall ultimate clinical utility of absolute MBF measurements. Also of potential interest in the future may be the use of 4D parametric image reconstruction, allowing for the reconstruction of parametric images from cardiac studies directly from sinograms,^32^,^33^ which potentially allow motion compensation along with a better estimation of the kinetic parameters than the traditional indirect approach of using frame-by-frame reconstruction and curve fitting to regional time-activity curves.

## Limitations

Our study has some limitations, with the foremost being that we analyzed the results of only 21 patients without stress MBF (and thus CFR) in this small study. However, as the image data originate from the same raw sinogram data, the effect of intra-subject biological variation was removed. Over these 21 patients, we showed a minor influence of BPL reconstruction in the automated quantification of MBF. Furthermore, rest perfusion imaging for sarcoid diagnosis is a niche application of MBF quantification, and efforts should be extended to investigate the effects of the BPL reconstruction with stress-rest studies, and any potential effect on the variation of the myocardial flow reserve (MFR) ratio. Although we expect the low coefficient of variation between reconstructions to be independent of flow rate, we currently lack the data to validate this claim. Furthermore, resulting image quality was not assessed in this work, and such an assessment of the blurring of high *B* value images using the BPL algorithm may be useful for identification of perfusion defects. A subjective imaging score by a trained physician could be used to visually compare the static [13N]-NH3 reconstructions in terms of their image quality rather than solely quantification of MBF. We have shown that for MBF calculations in our dataset, OSEM and FBP datasets are equivalent and produce comparable values, as has been shown by previous cardiac studies using low count density data in [15O]-H_2_O and [18F]-FDG studies,^34^,^35^ and at-rest MBF values (<3.4 mL·g^−1^·minute^−1^) from [13N]-NH3 studies.^17^ However, for our datasets, we now extend this equivalency to include PSF modeling via the BPL reconstruction algorithms investigated in this work as these algorithms begin to gain more clinical availability and prevalence.

## Clinical Relevance

New PET reconstruction algorithms such as the BPL technique which allow for effective convergence of image accuracy while also suppressing noise through the use of a penalization factor are becoming more commonplace in clinical PET imaging. However, the use of these reconstruction techniques have not yet been assessed in the quantification of MBF. There is a requirement to demonstrate that quantification of MBF is at least as good using BPL reconstruction as the current gold standard, which due to linearity reasons at highly changing activity concentrations in our case is an FBP reconstruction.

## New Knowledge Gained

Our study of MBF in 21 patients undergoing dynamic rest [13N]-NH3 imaging showed that using a PET reconstruction algorithm that runs to effective convergence with noise suppression, such as the BPL algorithm employed in this work, does not lead to significant differences in the quantification of rest MBF. We have also identified that the BPL algorithm with a *B* value of 300 gives the same level of noise in the image as a standard clinically used OSEM algorithm.

## Conclusion

Our work from this study shows that the effects of employing a BPL reconstruction in [13N]-NH3 cardiac PET data do not have a significant affect on the quantification of the rest MBF over all cardiac territories. The coefficient of variation over the entire reconstructions was found to be a maximum of 9.74%, and the use of the BPL algorithm with the increasing *B* value produced images with less image noise. Noise equivalence to standard OSEM reconstruction was achieved with a *B* value of 300.

## Abbreviations

OSEM: Ordered subsets expectation maximization
FBP: Filtered back projection
BPL: Bayesian-penalized likelihood
MBF: Myocardial blood flow
IDIF: Image-derived input function
CFR: Coronary flow reserve
