# Erratum to: Effect of Bayesian-penalized likelihood reconstruction on [13N]-NH3 rest perfusion quantification

**DOI:** 10.1007/s12350-017-0892-1

**Published:** 2017-05-02

**Authors:** Jim O’ Doherty, Daniel R. McGowan, Carla Abreu, Sally Barrington

**Affiliations:** 1 PET Imaging Centre, Division of Imaging Sciences and Biomedical Engineering, King’s College London, King’s Health Partners, St. Thomas’ Hospital, 1st Floor, Lambeth Wing, London, SE1 7EH United Kingdom; 2 Department of Oncology, University of Oxford, Old Road Campus Research Building, Oxford, OX3 7DQ United Kingdom; 3 Radiation Physics and Protection, Churchill Hospital, Oxford University Hospitals NHS Foundation Trust, Oxford, OX3 7LE United Kingdom

## Erratum to: J Nucl Cardiol 24(1):282–90 DOI 10.1007/s12350-016-0554-8

This article, which was not published as open access, is Wellcome Trust funded, which means that it has to be open access due to open access mandate. Thus the copyright line needs to be corrected as shown in this erratum. The original article was corrected.

